# Samuel Guthrie, Discoverer of Chloroform*Read before the Rochester Pathological Society, May 4, 1916.

**Published:** 1917-05

**Authors:** William V. Ewers

**Affiliations:** Physician Junior Staff Rochester General Hospital


### Samuel Guthrie, Discoverer of Chloroform.*

By DR. WILLIAM V. EWERS,

Physician Junior Staff Rochester General Hospital.

In these modern times history, both general and medical, is made very rapidly. Today a new discovery is announced; a new name is added to the honor role of medicine; tomorrow another will penetrate a little deeper into the secret of our science. In our haste to do homage to this latest claimant for honors we slight the hero of yesterday, and like the actor of a former generation, he is soon forgotten. We may make daily use of the discovery, but the name of the discoverer we no longer remember.

It is the function of medical history to keep alive the memory of these medical pioneers, and it is now and then interesting, if not instructive, to turn from things medical and consider their lives and works. It has been the reader's good fortune to spend his summers for a number of years in a lake village of northern New York where one of these medical pioneers lived and did his work. He was Dr. Samuel Guthrie, discoverer of chloroform. It has been the reader's intention for several years to write a sketch of his life, but for one reason or another it has been put off until the present time.

Dr. Guthrie was descended from a prominent Scotch family. The family were Covenanters, and one of them, James Guthrie, a minister of Sterling and son of the Laird of Guthrie, on account of his writings, was hanged at Edinburg on June 1, 1661. These religious persecutions forced the family to emigrate to the new world, where they first settled in Boston. From there they moved to different places in New England. In the pre-revolutionary days they were living in Lenox. The Doctor's grandfather took an active part in the events of the time, and when the war came he enlisted in the Revolutionary army with his two sons, Samuel and Joseph. Later Samuel, the father of our Doctor, studied medicine and settled in Brimfield, Mass., where he became prominent in his profession. Here the subject of this sketch was born in 1782. He was the eldest child of his father's first marriage. Nothing is known of his boyhood. What education he had he evidently received in Brimfield. We know that he studied medicine with his father, and when ready to practice removed to Scherburns, Chenango Co., N. Y., where his grandfather,

---

*Read before the Rochester Pathological Society, May 4, 1916.

James G. Guthrie, had moved in 1792. Whether his father's second marriage had anything to do with this move we do not know. His father in his will left him one dollar, Dr. Rush's Enquiries in five volumes, and a set of silver catheters. As the other children fared about the same, the bulk of the estate going to the widow, he probably was not slighted.

It was at Sherburne that he began the practice of medicine, and we are told that he was successful. That he was made of the right stuff the following incident seems to prove. Jenner had only recently announced the discovery of vaccination. Guthrie was evidently much impressed, and soon became a firm believer in the value of the preventative against small-pox. Being called to attend a case of the disease and wishing to prove to the community the value of vaccination, he vaccinated his cousin, Sarah Guthrie, who had volunteered to nurse the patient; in whose room she also slept. Neither nurse nor Doctor contracted the disease. Though history does not state, I presume that the Doctor was also vaccinated. This vindication of the value of vaccination seems to have increased the Doctor's reputation for we are told that from this time on his advance was rapid.

It was while living at Sherburne that he became interested in manufacturing and experimenting with gunpowder. This interest never flagged during the rest of his life; and there were occasions when it came near causing his death, as he was involved in several severe explosions. In one of the last letters he ever wrote, he told his daughter that he was experimenting with gun-cotton.

In 1804 he married Sybil Sexton of Smyrna. Mrs. Guthrie was born at Somers, Conn., in 1788, and moved to Sherburne with her parents in 1795. She seems to have been a woman of strong character, well fitted to be the mate of such a person as the Doctor. Four children were born of this marriage, two sons and two daughters.

The next we hear of Guthrie is that during the winter of 1810-11 he attended medical lectures at the P. & S. New York. During the war of 1812 he was examining surgeon for the army. Where his duties took him at this time we do not know. The next we hear of him is that in January 1815 he attended medical lectures at the U. of Penn. While there he kept a journal that contained descriptions of what he saw, and in it, we are told, he did not hesitate to criticise his teachers. Unfortunately up to the present I have been unable to find this journal. I have no doubt that it contains much that would be of interest at the present time. It consisted of 275 closely written pages, some containing more than 200 words to the page. After the war of '12 Guthrie had established a vinegar factory at Sackett's Harbor, N. Y., for supplying Madison Barracks', the army post at that point. In 1817 he removed there with his family, locating about a mile from the village on Mill Creek at a place called Jewittsville. Mill Creek furnished sufficient water power for his needs; that is, his laboratory, powder and vinegar factory, and alcohol distillery.

His activities were not limited to his factories and laboratory. He also practiced farming and horticulture, and for a short time engaged in the practice of medicine in his new home. He set out a vineyard that evidently thrived, as it became one of the show places of the neighborhood, and was visited by people from far and near. These visitors became such a nuisance that the Doctor finally had the vineyard dug up, and even the stone wall removed. Whether the bother of these visitors was the only consideration that prompted this act I am inclined to doubt; as Sackett's is rather far north for grapes to do well, and as far as I know there are no vineyards in those parts at the present time.

The next thirteen years must have been busy ones for the Doctor. He probably devoted his time to his different business pursuits. At any rate he was not publishing any of his experiments. In 1826 he began the manufacture of priming powder. This soon gave him a reputation with sportsmen, and his powder was extensively used in this country and Canada. His manufacturing must have been on a rather extensive scale for those days and that place, as Professor Stillman, in speaking of the chemicals that "Mr. G." had sent, says that "Mr. G. has made, and generally by processes peculiar to himself, one hundred and twenty thousand gallons of vinegar and one hundred thousands of alcohol," and in another place states that Guthrie had manufactured within three or four years twelve hundred pounds of chlorate of potassia, and this enormous quantity is partly accounted for by the fact that he sells one thousand ounces per annum of his priming powder, of which the chlorate of potash is an indispensable ingredient. With all these activities Guthrie did not neglect working in his laboratory, for in another article he tells us "A series of experiments was commenced at my laboratory" on the making of sugar from potato starch. He later induced a friend of his, a Captain Potter, to undertake the manufacture of the sugar, on a large scale. The experience he gained in this manufacture he communicates to the American Journal, and in a true scientific spirit tells how he did it and where he thinks improvement could be introduced. Evidently neither Guthrie nor his friend knew that they were making glucose, instead of cane sugar. Their product was a thick syrup, and they were unable to produce it in crystalline form unless they made it in lead retorts, which Guthrie claimed contaminated the sugar.

It was probably about this time, or a little earlier, that he invented the percussion lock. Dr. Guthrie fired the cannon at Madison Barracks with some of his percussion powder, using a hammer to ignite the powder. An officer who witnessed the experiment laconically remarked that soldiers could not be expected to go to war carrying hammers. This remark evidently sunk in, for when the doctor went home, he made a percussion lock. On the seal of the Jefferson County Historical Society there is a representation of Guthrie firing the cannon.

For medical men the most interesting of Guthrie's discoveries was that of chloroform. It is this discovery that entitles him to a place in works on medical biography. It is one of the paradoxes of nature that the man who gave the world percussion powder, a substance that has added to the miseries of the world, should be the discoverer of chloroform, which has helped to relieve the sufferings of mankind.

Like many other medical discoveries, chloroform was first made by three investigators, working independently, in different parts of the world. They were Soubeiran of France, Liebig of Germany, and Guthrie of America. The priority of Guthrie's claim has been well substantiated. The evidence has been well sifted by the Chicago Medical Society, which a number of years ago investigated the subject and made a report favoring the claim of Guthrie. Ossian Guthrie, a grandson of the Doctor, collected much evidence, which he published in a pamphlet on the life of the Doctor. Soubeiran's article on ether bichlorique appeared in the "Annales de Chimie et Physique" for October 1831 and in the "Journal de Pharmacie" for January 1832, of which journal Soubeiran was co-editor. Liebig has shown that on account of the political disturbances in France at that time the "Annales de Chimie et Physique" did not appear until January 1832. This is further proven by the fact that it contains the meteorological records for the whole month of October 1831, and an article dated January 1832. Liebig claims to have published an article in Poggendorff's Annalen of November 1831, on the decomposition of alcohol by chlorine, in which he states that a new chloride of carbon is formed (1) by the action of aqueous alkalies on chloral, (2) by the distillation of alcohol with excess of bleaching powder, and (3) by distilling bleaching powder with acetone. Guthrie's first article appeared in the first part of volume XXI, page 64 of the American Journal of Science and Arts, the editor of which was Professor Sillman of Yale. The article is entitled ''A new mode of preparing a spirituous solution of chloric ether.'' The first part of volume XXI was issued in October 1831, the journal being issued quarterly the first of April, July, October and January. The April and July numbers were bound in one volume and the October and January ones in another. So when volume XXI was issued it was dated January 1832. All the articles in this first part that are dated, bear dates early in the summer of 1831. The date of Guthrie's article is not given, but the one just preceding it is dated July 1831, and the second article after it, July 2, 1831. From this it has been assumed that Guthrie's article was probably written during this month.

The only article following Guthrie's that bears an earlier date than July is one of June 25, 1831. The editor states, in a footnote, that this was received too late for insertion in the last number (that of July 1831). The parenthesis is that of the editor. The only other article following Guthrie's that is dated, is dated August 1831. In this first article Guthrie makes the statement that ''During the last six months, a great number of persons have drunk of the solution of chloric ether in my laboratory.'' This places the discovery of chloroform early in the year 1831. In the second part of this volume there is a letter from Guthrie dated Sackett's Harbor Sept. 12, 1831, in which among other things he says that he is sending this day Professor Sillman the chemicals enumerated, among which he mentions a bottle and phial containing alcoholic solution of chloric ether. The editor adds that they have been received.

So far as I know, the only statement that Guthrie made in regard to this subject is in the letter which he wrote to his daughter Harriett, dated Feb. 9, 1848. This is the so-called ''Chloroform letter.'' He wrote as follows: ''I could have made a fortune if I had gone to New York as I was urged last fall; by making sweet whiskey, which you remember taking when suffocated with charcoal. You see it is called chloroform, and the newspapers are beginning to give me the credit of discovering it. I made the first particle that was ever made, and you are the first human being that ever used it in sickness. This is likely to prove the greatest discovery in medicine the world ever saw. By breathing it a few seconds, the person falls apparently into a sweet sleep; when breasts, legs and arms may be cut away, painful labors ended, and all without pain or injury.''

(To be concluded in June issue).

